# Frailty, falls and poor functional mobility predict new onset of activity restriction due to concerns about falling in older adults: a prospective 12-month cohort study

**DOI:** 10.1007/s41999-023-00749-2

**Published:** 2023-02-05

**Authors:** Toby J. Ellmers, Kim Delbaere, Elmar C. Kal

**Affiliations:** 1 Centre for Vestibular Neurology, Department of Brain Sciences, Imperial College London, London, UK; 2 Centre for Cognitive Neuroscience, Department of Clinical Sciences, Brunel University London, Uxbridge, UK; 3 Falls, Balance and Injury Research Centre, Neuroscience Research Australia, Randwick, NSW Australia; 4 Australia and School of Population Health, University of New South Wales, Kensington, NSW Australia

**Keywords:** Fear of falling, Concerns about falling, Activity avoidance, Deconditioning, Falls prevention

## Abstract

**Purpose:**

Concerns about falling are common in older adults and often cause activity restriction. This can lead to physical deconditioning, falls and social isolation. However, not every concerned older adult will restrict their activities. This 12-month longitudinal study investigated the physical and psychosocial factors that predict the new onset of activity restriction due to concerns about falling in older people.

**Methods:**

Participants were 543 older adults (*M*_age_ = 80.3 ± 4.4 years, range: 75–98) who did not report activity restriction due to concerns about falling at Timepoint-1 (negative response to the following question: “Do concerns about falling stop you going out-and-about?”). Participants completed a battery of physical and psychological assessments at Timepoint-1. Using binary logistic regression, we then assessed which of these variables predicted whether participants reported having started restricting their activity due to concerns about falling at the 12-month follow-up (Timepoint 2).

**Results:**

10.1% of the sample started to restrict activity due to concerns about falling at Timepoint 2. Three key predictors significantly predicted activity restriction group status at 12-month follow-up: greater frailty at Timepoint-1 (Fried Frailty Index; OR = 1.58, 95% CI 1.09–2.30), experiencing a fall between Timepoint-1 and 2 (OR = 2.22, 95% CI 1.13–4.38) and poorer functional mobility at Timepoint-1 (Timed up and Go; OR = 1.08, 95% CI 1.01–1.15).

**Conclusions:**

Frailty, experiencing a fall and poorer functional mobility all predicted the onset of activity restriction due to concerns about falling. Clinicians working in balance and falls-prevention services should regularly screen for frailty, and patients referred to frailty services should likewise receive tailored treatment to help prevent the development of activity restriction due to concerns about falling.

## Key summary points

**Aim:**

To investigate the physical and psychosocial factors that predict the new development of activity restriction due to concerns about falling in older people.

**Findings:**

Our findings show that frailty, experiencing a fall and poorer functional mobility all predict the new development of activity restriction.

**Message:**

Clinicians working in frailty services should refer patients to services for activity restriction due to concerns about falling.

## Introduction

Concerns (or, “fear”) about falling are reported by up to 50% of older people [1, 2]. Traditional conceptualisations have viewed concerns about falling as a universally negative construct. This is largely due to research that has reported its association with reductions in physical and mental wellbeing [2–4]. However, emerging evidence suggests that a certain level of concern about falling may, for some individuals at least, serve an adaptive purpose–particularly if this represents a realistic appraisal of one’s risk of falling [5, 6]. Problems arise if the level of concern experienced outweighs the individual’s *actual* risk of falling [5], or if it leads to individuals avoiding activities outside of the home [1, 2, 7]. Such activity restriction can trigger a debilitating spiral of physical deconditioning, falls, social isolation, diminished confidence, and a loss of one’s sense of self [4, 6–9]. A better understanding of the factors that predict activity restriction due to concerns about falling is required to develop effective strategies to prevent it.

Not every older person who is concerned about falling will go on to develop activity restriction. For example, activity restriction is only reported by between 10 and 50% of older people with such concerns [1, 10, 11]. Activity restriction due to concerns about falling was reported to be more likely to occur in women, those who were older, and those who had fallen. Other studies highlight associations between activity restriction and poor physical functioning [12, 13], as well as psychological factors such as depression, general self-efficacy and cognitive function [12–14]. Yet, these factors have not been consistently replicated across the literature (e.g. [12, 14]). Moreover, the cross-sectional nature of this previous research means that we are unable to assess if previously identified factors *predict* activity restriction, or if they are a consequence of it.

The only previous longitudinal study on this topic found self-reported unsteadiness to be a strong predictor of future activity restriction due to concerns about falling [15]. However, as this study focused primarily on postural instability and balance, the predictive value of other physical (e.g., frailty, physical independence, etc.) and psychological factors (e.g., resilience, general self-efficacy, etc.) remains unclear. To inform the development of effective clinical interventions, we therefore conducted a longitudinal study to test whether specific physical and psychological factors predict the new onset of activity restriction due to concerns about falling in community-dwelling older people over a 12-month period.

## Methods

### Sample

The data reported herein were collected as part of the Community Ageing Research 75 + (CARE75 +) study [16]. The CARE75 + is a longitudinal cohort study conducted in community-dwelling older people living in England aged ≥ 75 years. As part of this protocol, detailed information was collected on participants’ demographic, health and social circumstances over multiple time points. The present analysis includes all participants enrolled in this study who had no diagnosed neurological disorder, were not registered blind, scored ≥ 18 on the Montreal Cognitive Assessment (indicating an absence of major cognitive impairment) [17], and who had baseline (T1) and 12-month follow-up (T2) data collected prior to October 2019.

Data were analysed for 614 participants, who had a mean age of 81.7 years (SD = 4.63, range = 75–98 years). Whilst this was a pragmatic sample size, we considered *n* = 500 as the minimum required to conduct a logistic regression with 11 predictors, and the conservative estimation that between 10 and 20% of participants would develop activity restriction [1, 10].

All participants provided written informed consent. The CARE75 + study was approved by the NRES Committee (Yorkshire and the Humber—Bradford Leeds; 14/YH/1120), and the specific analysis reported herein was approved by Brunel University London’s College of Health and Life Sciences Research Ethics Committee (24,062-NER-Jul/202–26,428-1).

### Data collection

The CARE75 + protocol collects a range of demographic information. For the present research, we extracted age, gender, body mass index, educational qualifications, self-reported previous falls in the past 12 months i.e., if participants had experienced a fall between T1 and T2, as self-reported at T2), as well as a number of prescription medications from the available demographic data.

Based on previous cross-sectional and qualitative research investigating physical and psychological factors associated with activity restriction due to concerns/fear about falling [6, 12–14], we also extracted measures of frailty, independence in performing activities of daily living (ADL), functional mobility, cognitive function, depression, resilience and self-efficacy from the CARE75 + database. Frailty was assessed using the Fried Frailty Index [18], a phenotype model of frailty that categorises participants based on five physical characteristics (slow walking speed, weight loss, exhaustion, weak grip strength, low energy expenditure). Higher scores reflect greater frailty. The Barthel Index [19] was used to measure independence in performing basic ADL (e.g., bathing, dressing, feeding, grooming, toilet use, etc.), whilst the Nottingham Extended ADL scale [20] was used to assess instrumental ADL (e.g., everyday activities in the domains of mobility, kitchen, domestic and leisure). Higher scores on both scales reflect greater ADL independence. The Timed up and Go (TuG) test [21] was used to assess functional mobility. This involves timing a participant whilst they stand up from a chair, walk 3 m, turn around and then walk back to the chair and sit back down. Grip strength was also recorded using a Jamar dynamometer. As per the Cardiovascular Health Study [18], the mean score of three attempts using the dominant hand was recorded. Cognitive function was measured using the Montreal Cognitive Assessment [17]. Resilience was assessed using the Brief Resilience Scale [22]. This features items such as “I have a hard time making it through stressful events” and “I tend to take a long time to get over set-backs in my life”. The General Self-efficacy Scale [23] features items such as “I can always manage to solve difficult problems if I try hard enough” and “I can remain calm when facing difficulties because I can rely on my coping abilities”, and was used to measure self-efficacy. Finally, the 15-item Geriatric Depression Scale [24] was used to measure depression.

As reported in the CARE75 + protocol [16], each of these measures was carefully selected for inclusion in the protocol based on possessing the “[…] necessary validity, reliability and responsiveness to enable both applied epidemiological investigation and randomised trial evaluation of future interventions” (p. 2). Please refer to the CARE75 + protocol [16] for further information about the data collection protocol, or any of the specific measures collected.

### Dependent variable

In line with previous research [1, 11, 15], we used a single-item measure to assess concerns/fear-related activity restriction. Participants were categorised as having ‘activity restriction due to concerns about falling’ if they answered ‘Yes’ to the following question: “*Do concerns about falling stop you going out-and-about?”* Whilst more thorough assessments of activity restriction/avoidance do exist (e.g., the 17-item Survey of Activities and Fear of Falling in the Elderly [25]), from our experience these are rarely used in clinical practice due to the length required for administration. We, therefore, deemed a single-item measure of activity restriction to be the most clinically relevant.

### Statistical analysis

For this analysis, we first made an initial selection of older adults who did not report already restricting their activities due to concerns about falling at T1. Within this group, we then assessed which of the older adults would go on to develop concerns about falling-related activity restriction at a 12-month follow-up (T2). We then compared these ‘activity restriction’ and ‘non-activity-restriction’ groups on key characteristics, including demographics, measures of ADL independence, mobility/balance, and cognitive factors (see Table 1), using Mann–Whitney U tests or chi-square tests, as appropriate.

**Table 1:** Characteristics for both participant groups

	No activity restriction T2 (*N* = 488)	Activity restriction T2 (*N* = 55)	*p*-value
	Mean ± SD (range)	Mean ± SD (range)	
**Demographics (T1)**			
Age in years	80.2 ± 4.4 (75–98)	81.5 ± 4.5 (75–92)	*p* = 0.026
Gender (female; *N* (%))	246 (50%)	35 (64%)	*p* = 0.063
Body Mass Index (kg/m^2^)^a^	27.3 ± 4.5 (17.2–52.9)	27.2 ± 5.1 (17.4–40.1)	*p* = 0.993
Educational qualification (*N* (%))^b^			*p* = 0.074
None	218 (46%)	34 (63%)	
Below degree level	192 (41%)	14 (26%)	
University degree or higher	63 (13%)	6 (11%)	
**Independence/general functioning (T1)**			
Fried Frailty Index (median ± IQR (range)) (0–5)	1 ± 2 (0–5)	2 ± 1 (0–5)	*p* < 0.001
Nottingham Extended ADL scale (0–66)	59.2 ± 7.9 (24–66)	52.2 ± 11.5 (17–66)	*p* < 0.001
Barthel Index (0–20)	19.6 ± 1.1 (9–20)	18.8 ± 2.5 (6–20)	*p* = 0.003
Prescription medication use (no.)	5.3 ± 3.9 (0–21)	7.4 ± 3.6 (0–19)	*p* < 0.001
**Mobility and balance (T1)**			
Faller status (fell in between T1-T2; *N* (%))^c^	106 (22%)	24 (44%)	*p* < 0.001
Timed-up-and-Go^d^ (seconds)	11.9 ± 4.6 (4.6–40)	16.2 ± 6.5 (7–32)	*p* < 0.001
Dominant Hand Grip Strength (kg/f)^e^	21.7 ± 9.3 (0–48)	16.9 ± 8.3 (2–36)	*p* < 0.001
**Cognitive/Psychological function (T1)**			
Montreal Cognitive Assessment (0–30)	26.2 ± 2.8 (18–30)	25.4 ± 3.2 (18–30)	*p* = 0.080
Geriatric Depression Scale (0–15)	1.6 ± 1.9 (0–12)	2.8 ± 2.4 (0–11)	*p* < 0.001
Brief Resilience Scale^f^ (1–6)	3.9 ± 0.6 (2–5)	3.7 ± 0.7 (2–5)	*p* = 0.114
General Self-efficacy Scale^g^ (10–40)	33.5 ± 4.1 (18–40)	32.3 ± 4.1 (24–40)	*p* = 0.047

^a^64 missing responses for ‘no activity restriction’ group, 10 missing responses for ‘activity restriction’ group

^b^15 missing responses for ‘no activity restriction’ group, 2 missing response for ‘activity restriction’ group

^c^2 missing responses for ‘no activity restriction’ group

^d^13 missing responses for ‘no activity restriction’ group, 3 missing responses for ‘activity restriction’ group

^e^12 missing responses for ‘no activity restriction’ group, 2 missing responses for ‘activity restriction’ group

^f^18 missing responses for ‘no activity restriction’ group, 2 missing responses for ‘activity restriction’ group

^g^29 missing responses for ‘no activity restriction’ group, 1 missing response for ‘activity restriction’ group

NB: all comparisons either concerned Mann–Whitney *U* tests or chi-square tests, as appropriate

Using multivariable logistic regression (performed using Jamovi v.1.6.23), we further analysed which factors were (most) predictive of later activity restriction group status. Based on cross-sectional and qualitative research investigating physical and psychological factors associated with concerns about falling-related activity restriction [6, 12–14], we used the following predictors (as collected at baseline (T1)): Gender, age in years, frailty (Fried Frailty Index), independence of ADL (Nottingham Extended Activities of Daily Living^1^), faller status between T1 and T2, functional mobility (TuG), grip strength, cognition (Montreal Cognitive Assessment), depression (Geriatric Depression Scale), resilience (Brief Resilience Scale), and self-efficacy (General Self-efficacy Scale). All variables were entered simultaneously. Model fit was evaluated using Nagelkerke R^2^. There were no multicollinearity issues (variance inflation factors ≤ 2.3, tolerances ≥ 0.4). The assumption of linearity for continuous variables was met as evidenced by the absence of significant ‘predictor * ln(predictor)’ interactions.^2^ Alpha was set at 0.05 for all analyses.

### Data availability statement

This study used third-party data made available under licence that the authors do not have permission to share. Requests to access the data should be directed to the CARE75 + Research Team at https://www.bradfordresearch.nhs.uk/care75/data-request/.

## Results

In total, 614 older adults met the inclusion criteria. Of these, 71 were excluded as they already reported to restrict their daily activities due to concerns about falling at T1. As a result, 543 older adults were included in our analysis. Of these, 55 older adults reported to have started to restrict activity due to concerns about falling at T2 (10.1% of the overall sample), whilst 488 people reported (still) not restricting their activities (89.9%). Detailed characteristics of both groups are presented in Table 1. Overall, the ‘activity-restriction’ group scored worse on measures of frailty, ADL independence, falls and functional mobility. Whilst some differences in cognitive/psychological function were evident, these were primarily restricted to differences in (i.e., higher in the activity restriction group) depression scores.

The results of the logistic regression are summarised in Table 2. As can be seen, three key predictors were found to significantly predict activity restriction group status at 12-month follow-up: frailty, faller status and functional mobility. That is, those who scored higher on the Fried Frailty Index at T1 had significantly greater odds of restricting activity due to concerns about falling at T2 (OR: 1.580, 95% CI 1.087–2.297, *p* = 0.017). Similarly, having experienced a fall between T1 and T2 was significantly associated with significantly higher odds of subsequently reporting to have started restricting activities at T2 (OR: 2.219, 95% CI 1.125–4.375, *p* = 0.021). Finally, poorer functional mobility (i.e., slower TuG scores) seemed to also independently associate with greater odds of avoiding activity at follow-up (OR: 1.076, 95% CI 1.005–1.152, *p* = 0.036). None of the other variables significantly predicted the new onset of activity restriction due to concerns about falling.

**Table 2:** Results of logistic regression predicting new-onset activity restriction due to concerns about falling at T2

	*p*	Odds Ratio [95% CI]
Intercept		
**Demographics (T1)**		
Age (in years)	0.816	0.991 [0.919, 1.069]
Gender (reference = male)	0.560	1.294 [0.545, 3.074]
**Independence / general functioning (T1)**		
ADL-independence (NEADL scale)	0.733	0.992 [0.945, 1.041]
Frailty (FFI)	**0.017**	1.580 [1.087, 2.297]
**Mobility and balance (T1)**		
Faller Status (reference = non-faller)	**0.021**	2.219 [1.125, 4.375]
Functional Mobility (TuG)	**0.036**	1.076 [1.005, 1.152]
Dominant Hand Grip Strength (kg/f)	0.948	1.002 [0.948, 1.059]
**Cognitive/Psychological function (T1)**		
Cognitive function (MoCA)	0.776	0.984 [0.880, 1.100]
Depression (GDS)	0.613	1.048 [0.873, 1.259]
Resilience (BRS)	0.925	0.970 [0.511, 1.840]
Self-Efficacy *(GSES)*	0.627	1.023 [0.932, 1.123]

*NB* Nagelkerke *R*^2^ = 0.211; *χ*^2^(10) = 51.2, *p* < 0.001; AUC = 0.80, Specificity = 0.73, Sensitivity = 0.76, cut-off: 0.09

*BRS* brief resilience scale, *FFI* fried frailty index, *GDS* geriatric depression scale, *GSES* general self-efficacy scale, *MoCA* montreal cognitive assessment, *NEADL* nottingham extended activities of daily living scale, *TUG* timed-up-and-Go

## Discussion

Previous cross-sectional research has highlighted associations between activity restriction due to concerns about falling and various demographic, physical and psychological factors [12–14]. However, the cross-sectional nature of this work means that we are unable to assess if these factors *predict* activity restriction, or if they are a consequence of it. This limits our ability to design effective interventions. To overcome the limitations of this previous work, we investigated the factors that predict the new development of concerns about falling-related activity restriction in older people using a prospective cohort study. We found that 10% of participants developed activity restriction due to concerns about falling over a 12-month period. Our key findings show physical frailty at T1 (as assessed by the Fried Frailty Index [18]), experiencing a fall between T1 and T2, and poorer functional mobility at T1 (as assessed by the TuG) predict the new onset of activity restriction at T2.

Previous cross-sectional research has reported associations between concerns about falling-related activity restriction and frailty, falls, and functional mobility [1, 13]. However, our study is the first to demonstrate a predictive relationship between these factors via a longitudinal design. The present findings build upon the single previous longitudinal study which highlighted the role of poor (self-reported) balance in predicting the future development of activity restriction due to concerns about falling [15].

In contrast to previous cross-sectional studies on this topic (e.g. [1]), participants’ age was not a significant predictor of activity restriction. This could be due to the restricted age range within our sample (75 years and above), as reduced variance in age could limit its potential predictive power. In addition, both fall risk and frailty increase with age, so it could also be that such age-related changes in these factors explain earlier reports in which higher age was linked to greater activity restriction.

Previous cross-sectional research has also reported that certain psychological factors (namely depression, general self-efficacy and cognitive function) are associated with the development of activity restriction due to concerns about falling [12–14]. Whilst those who went on to develop concerns about falling-related activity restriction in the present research did significantly differ on a number of psychological variables at T1 (see Table 1), none of these variables significantly predicted group status. This suggests that the associations reported in this previous cross-sectional work are likely due to these psychological factors being a consequence–rather than a cause–of activity restriction. Our results, therefore, predominantly emphasise the importance of physical factors when assessing an individual’s risk for developing activity restriction due to concerns about falling.

That said, based on our current results, we should not definitively rule out the role of (neuro-)psychological factors. For one, we used a broad measure of general cognition (MoCA), but it may well be that we need to include more sensitive assessments or more *specific* cognitive functions (e.g., executive function) in our prediction model–which we did not have access to. Further, psychological factors such as resilience, which is an individual’s ability to adapt to adverse life events, will only become relevant *if* such adverse events occur (i.e., if experiencing a fall). A recent conceptual model proposes that the recognition of one’s own risk for falls and injury–coupled with a lack of perceived control and low resilience–is a crucial step in the development of activity restriction due to concerns about falling [6]. Indeed, a multicomponent cognitive behavioural intervention has been found to both enhance resilience and reduce activity restriction due to concerns about falling [26]. Therefore, future research should determine if *interactions* between (changes in) faller status and factors such as resilience/self-efficacy predict the onset of concerns about falling-related activity restriction.

### Clinical implications

These findings build upon previous cross-sectional work and identify numerous potentially modifiable factors that predict the new onset of activity restriction due to concerns about falling. First, we recommend that patients referred to frailty services should be directed towards tools (e.g., educational materials) or services (e.g., balance exercise interventions) that are explicitly designed to reduce concern-related activity restriction, in conjunction with strategies designed to target frailty itself. Second, we recommend that frailty is regularly screened in balance and falls prevention services given both the previous associations between frailty and falls [27], and the current finding that frailty predicts activity restriction due to concerns about falling. While our data do not directly suggest an important role of specific psychological factors, cognitive behavioural interventions have been found to reduce activity restriction due to concerns about falling, so probably should be considered as well [26]. Finally, our findings also show that experiencing a fall predicts the development of activity restriction due to concerns about falling. We therefore recommend that, regardless of their frailty status, older people who are referred to rehabilitation services following a fall should also receive treatment designed to specifically prevent the development of activity restriction due to concerns about falling.

### Strengths and limitations of study

To our knowledge, this is the first prospective cohort study to investigate a full array of physical and psychological factors predicting the development of activity restriction due to concerns about falling. Another strength is that the sample is representative of the wider (75 years and older) community-dwelling older adult population, with participants’ ages ranging from 75 to 98 years (mean age of 81.7 years).

A main limitation of this work relates to the relatively small sample of participants who developed concerns about falling-related activity restriction over the 12-month period (10.1% of the sample; *n* = 55 participants). A previous longitudinal study reported that ~ 18% of older adults developed new onset of concern/fear-related activity restriction over a 12-month period [15]. The slightly lower number of people reporting activity-related restriction at T1 (~ 12%; those excluded prior to analysis) and at T2 (~ 10%) in the present study are likely due to the differences in the specific question used to assess activity restriction. For instance, the activity restriction question used in this previous study asked participants if they “ever limited their activities, for example, what they did or where they went”. In contrast, we asked participants if their concerns about falling stopped them from leaving their house–a more extreme, but perhaps less common, form of activity restriction. Relatedly, although such single-item assessments are common in both research (e.g. [1, 15]) and clinical settings to screen for activity restriction due to concerns/fear about falling, future studies could also use more elaborate assessments (e.g., the 17-item Survey of Activities and Fear of Falling in the Elderly [25]). This may also further enhance our understanding of the specific activities that frail individuals avoid, allowing for targeted therapeutic intervention.

## Conclusion

The study findings show that frailty, experiencing a fall and poorer functional mobility predict the development of activity restriction due to concerns about falling. Clinicians working in balance and falls-prevention services should regularly screen for frailty, and patients referred to frailty services should likewise receive tailored treatment to help prevent the development of activity restriction due to concerns about falling.

## Footnotes

1 We used this as our predictive measure of independence of ADL, rather than the Barthel Index, due to 74.8% of participants scoring the highest possible score (i.e., total independence) on the Barthel Index, compared to only 23.9% on the Nottingham Extended ADL scale.

2 The only exception where this assumption had been violated was the ‘resilience’ variable (*p* < .05). As the main results of our analyses were the same regardless of whether this variable was included or excluded, we decided to proceed with this variable included in the model.
